# Preparation, Characterization, and Leishmanicidal Assessment of Silver- and Dapsone-Loaded Niosomes Co-administration: *In Silico* and *In Vitro* Study

**DOI:** 10.34172/apb.42740

**Published:** 2024-12-05

**Authors:** Anna Etemadifar, Sina Bahraminejad, Abbas Pardakhty, Iraj Sharifi, Alireza Keyhani, Mehdi Ranjbar

**Affiliations:** ^1^Pharmaceutics Research Center, Institute of Neuropharmacology, Kerman University of Medical Sciences, Kerman, Iran.; ^2^Leishmaniasis Research Center, Kerman University of Medical Sciences, Kerman, Iran.; ^3^Neuroscience Research Center, Institute of Neuropharmacology, Kerman University of Medical Sciences, Kerman, Iran.

**Keywords:** Niosomes, Drug delivery, Combination therapy, Cutaneous leishmaniasis, Dapsone, Silver

## Abstract

**Purpose:**

Currently, there is a crucial need for alternative strategies to control leishmaniasis, which threatens more than 1 billion people worldwide. The simultaneous use of combination therapy and nanostructured lipid carriers aimed to assess the leishmanicidal activity of silver and dapsone niosomes co-administration *in vitro* and *in silico*.

**Methods:**

After preparing the niosomal formulations of dapsone and silver using the film hydration method, Span 40 and Tween 40/cholesterol with a 7/3 molar ratio was selected as the optimal formulation. Consequently, the arrays of experimental approaches were conducted to compare the anti-leishmaniasis efficacy of ready niosomes with amphotericin B and obtain a deeper understanding of their possible mechanisms of action.

**Results:**

Our findings showed higher potency of silver-loaded and dapsone-loaded niosomes co-administration compared to amphotericin B as a positive control group. The results of isobologram and combination index (CI) analyses confirmed the synergic potential of this mixture. This combination triggered anti-leishmanial pathways of macrophages, which promoted the expression level of Th1 cell-related genes, and the downregulated expression of the phenotypes related to Th2 cells. Furthermore, a high level of antioxidant and apoptotic profiles against the parasite provides a rational basis and potential drug combination for cutaneous leishmaniasis. Moreover, the outcomes of the molecular docking showed a high binding affinity between dapsone and iNOS, stimulating the immune response in Th1 direction.

**Conclusion:**

In general, the multifunctional leishmanicidal activity of dapsone and silver-niosomes co-administration should be considered for further *in vivo* and clinical studies.

## Introduction

 Leishmaniasis is a vector-borne infectious disease and an important public health concern, especially in tropical and semi-tropical regions.^1,2^ This potentially lethal disease spreads via the bites of phlebotomine sand fly species, which is caused by various *Leishmania* species.^3,4^ Leishmaniasis has a wide range of clinical manifestations, from skin lesions to progressive damage to vital organs, and it is categorized into three forms: visceral (VL), cutaneous (CL), and mucocutaneous leishmaniasis (ML). CL as the most common and VL (kala-azar) as the most fetal type of this disease are two main forms of leishmaniasis from 600 000 to 1 million and 50 000 to 90 000 new cases annually, respectively.^3,5-7^ Leishmaniasis is distributed in 98 countries of the world, and most of them are in Africa, the Middle East, South and Central America, and the Mediterranean region threatening more than 1 billion people. Cutaneous leishmaniasis in Middle Eastern countries like Iran is mainly caused by *Leishmania major* and *L. tropica.*^8,9^

 Historically, numerous drugs, such as pentavalent antimony compounds such as sodium stibogluconate and meglumine antimoniate formulations have been used for the treatment of leishmaniasis as a first line, and liposomal forms of amphotericin B, pentamidine, miltefosine, paromomycin, and azole antifungals were considered the alternatives if resistance occurs.^6,10,11^ Even though these treatments were found effective, all of these drugs cause several related limitations and adverse effects, such as high cost and toxicity, parenteral administration, long-term treatment, and drug resistance.^12-14^

 Therefore, to find new, less toxic therapeutic agents, nanotechnology has played a significant role via various nanodevices and nanomaterials.^15,16^ Besides, using drug combinations instead of monotherapy can prevent drug resistance and decrease adverse reactions.^17^ Recently, several new systemic drugs showed antileishmanial activities. Dapsone (DAP, 4,4-diamino-diphenyl sulfone) is a suitable substitute for the treatment of leishmaniasis by increasing cellular immunity via different cytokines and its anti-inflammatory properties.^18-20^

 Several findings demonstrate that metallic and metal oxide nanoparticles (NPs) such as titanium dioxide (TiO_2_), zinc oxide (ZnO), iron oxide (Fe_3_O_4_), silver (Ag), and gold (Au) can inhibit the growth of various parasites such as *Leishmania.*^21,22^ In addition, combining Ag-NPs with chemical drugs can be used as an appropriate alternative for toxic drugs providing lower toxicity and higher efficacy.^23^

 Over recent years, nano-vesicular systems such as niosomes, liposomes, transferosomes, pharmacosomes, and ethosomes have been employed as promising strategies for drug delivery systems. Niosomes as nonionic surfactant-based nanovesicles have oral, parenteral, and topical applications to obtain the proper therapeutic response for the treatment of leishmaniasis. Niosomes can prolong their incorporated drug release and their residence time in the skin (epidermis and stratum corneum). These biodegradable and biocompatible drug delivery systems can decrease systemic absorption of their entrapped drug which leads to reducing toxicity as the main reason for developing a niosomal system. Niosomes are preferable rather than liposomes in terms of their higher chemical stability and skin penetrability, and lower production cost.^24-29^

 The present study aims to prepare the niosomal formulation of silver and dapsone to assess their antileishmanial activity on *L. major *promastigote and amastigote stages alone and in combination with each other using *in vitro* and *in silico *assays, and gene expression profiles. For this purpose, initially, the synthesized niosomal formulations were characterized, and the encapsulation efficiency and *in vitro* release study were conducted.

## Materials and Methods

###  Materials

 Amphotericin B as a drug of choice was purchased from Health Biotech ltd. India as the water-soluble intravenous infusion vials. Dapsone and silver were obtained from Merck, Germany. Sorbitan esters (Span^®^), their PEGylated derivatives (Tween^®^), and cholesterol were supplied by Fluka, Switzerland. All chemical reagents were employed without any purifications and prepared from Merck, Germany.

###  Preparation of niosomal formulations

 In the present study, a film hydration assay was utilized to synthesize niosomes. Briefly, the lipid phase which consists of nonionic surfactant (Span 40 and Tween 40)/Cholesterol was dissolved in 5 mL chloroform with specified molar ratios (7/3). Next, a rotary evaporator (Büchi Labortechnik AG, Switzerland) was used to remove organic solvent at 65 °C. The resultant lipid layer was kept in a vacuum desiccator overnight for eliminating the trace organic solvent remaining. To prepare Ag-loaded niosomes, the resultant dried lipid film was hydrated with 5 mL of Ag solution (distilled water) at 65 °C for 30 minutes. Then, smaller niosomes were obtained using a sonicator (Sonopuls HD-3200, Germany) by sonicating the hydrated niosomes with a frequency of 20 kHz for 5 minutes. Similarly, 5 mL of dapsone solution (acetone) was employed to synthesize dapsone-loaded niosomes. The final concentration of Ag and dapsone in their resulting niosomal suspension was set to 1% w/v. The resultant niosomes were stored at refrigerator temperature for further assessment.

###  Characterization of niosomes

 The formation of vesicles, the estimated number of vesicles, their different shapes (MLV, SUV, round, tubular, etc.), the presence of vesicle aggregation, constituent cholesterol crystals or surfactant particle or droplet separation, and phase separation were investigated using optical microscopy HFX-DXZ (Nikon, Japan). Some micrographs were prepared using a camera-attached optical microscope (10 × 40 magnifications).

 The static laser light scattering technique was used to measure the particle size distributions of vesicles via a Malvern Nano ZS light scattering apparatus (Worcestershire, Malvern Instruments Ltd., UK). Size distribution parameters and derived diameters were calculated from the fundamental size distribution by using Sizer-2000E software. To evaluate the Vesicle stability, vesicle size and the morphology of MLVs were determined in 1 week, 1, 3, and 6 months (stored in a refrigerator) after preparation. Measurement was carried out using a 100 mm focal length lens, which was capable of measuring vesicles in the 0.1-100 μm size range.

 The Fourier-transform infrared (FT-IR) spectra were obtained using the Shimadzu Varian 4300 spectrophotometer and the IR absorption spectra were recorded in the region of 400-4000 cm^-1^ and then compared with references.

###  Encapsulation efficiency determination

 The determination of λ_max_ for dapsone in ethanol 96% was conducted by scanning the fresh dapsone solution (40 µg/mL) between 200 to 400 nm. To separate non-entrapped dapsone niosomes from encapsulated ones, the vesicle suspensions were centrifuged at 20 000 rpm for 15 minutes at 25 ˚C. The amount of dapsone in the supernatant and also in the vesicles was analyzed after disrupting the niosomes with ethanol 96%. The same procedure was performed for silver niosomes. The encapsulation efficiency (% EE) was determined using the following equation:


(Eq. 1) $$\%\;EE=\left(C_{p}/C_{T}\right)\times 100$$

Where C_p_ is the concentration of the drug in the vesicle and C_T_ is the initial drug concentration added to the formulation.

###  In vitro drug release

 A vertical glass Franz-type diffusion cell with an active surface area of 1.5 cm^2^ and a receptor phase volume of 15 ml was used for *in vitro* release study of dapsone from niosomes at 37 ± 1 °C. Cellulose acetate dialysis membrane (Visking tube, MW cut-off 12000 D) was used as a barrier between donor and receptor compartments of the diffusion cell. Before the diffusion experiments, the acetate cellulose dialysis membrane was soaked in normal saline for 24 hours. The receptor compartment was filled with the receptor phase, which contains 60% ethanol at 96° and 40% phosphate buffer that the pH fixed at 7, and the donor compartment with 1 mL vesicular dispersion of dapsone. The medium in the receptor compartment was magnetically stirred at a rate of 100 rpm. Samples with the volume of One ml were withdrawn at fixed time intervals from the receptor compartment in 0-400 minutes and replaced with a similar volume of fresh receptor phase and the permeated drugs concentrations were measured by UV spectrometer S-3100 SCINCO.

###  Parasite and macrophage cell lines

*Leishmania major* strain (MRHO/IR/75/ER) was kindly provided by Leishmaniasis Research Center, Kerman University of Medical Sciences, and seeded in Novy-MacNeal-Nicolle medium. RPMI-1640 (Biosera, France), enriched with 100 μg/mL streptomycin,100 IU/mL of penicillin, and 10 % v/v heat-inactivated fetal bovine serum (FBS) (Gibco, Germany) was used to culture promastigotes.

 Pasteur Institute of Iran (Tehran) provided the cell line of J774 - A1 ATCC ® TIB-67^TM^ murine macrophages. Cells were incubated at 37 °C, and 5 % CO_2_ using Dulbecco’s modified eagle’s medium (DMEM), supplemented with 10% heat-inactivated FBS and 1% penicillin and streptomycin.

###  Anti-promastigote assay

 Evaluation of the antiparasitic activity of the prepared niosomes and their combination compared to amphotericin B was conducted against the promastigote form of *L. major *using MTT assay. For this purpose, 90 μL of the *Leishmania* parasites (2 × 10^5^ promastigotes/mL) from the logarithmic growth stage, and 10 μL of various concentrations (5-100 μg/mL) of niosomal formulation of silver (NA) and dapsone (ND), their combination (NA + ND), and amphotericin B (Amp B) as a positive control group was shifted to each well of 96-well microplate and placed at 25 °C ± 1 °C for 72 hours. Subsequently, 20 μL of prepared MTT (Sigma Chemical Co., St. Louis, Mo.) solution (5 mg/mL) was added to each well, and then, the promastigotes were incubated at a similar condition for 3 hours. 100 μL of promastigotes containing no drug is considered a negative control. Finally, after adding 100 μL of DMSO to each well, OD absorbance was obtained at 490 nm using an enzyme-linked immunosorbent assay (ELISA) reader (BioTek-ELX800). All the experiments were conducted in triplicate.

###  Anti-intramacrophage amastigote assay

 In order to assess the anti-amastigote activity of prepared niosomes, initially, 200 μL of J774-A1 (5 × 10^5^ cells/mL) was cultured in DMEM medium enriched with 10% heat-inactivated FBS using sterile glass slides in 6-chamber plates. After 24 hours of incubation at 37 °C in 5 % CO_2_, 200 μL of *L. major* parasites (5 × 10^6^ promastigotes/mL) in the stationary growth phase were added to the adhered macrophages at a ratio of 10:1 parasite/macrophage and then, the infected macrophages were incubated overnight, similarly. Following washing out free parasites with PBS, intramacrophage amastigotes were exposed to different concentrations (25-100 μg/mL) of prepared niosomal formulation of silver (NA) and dapsone (ND), their combination (NA + ND), and amphotericin B (Amp B) as a positive control group. Then, all the samples were kept under the same conditions for 72 hours. All the cultures were conducted in triplicate. Afterward, the light microscope was used to count the number of amastigotes by examining 100 cells in each sample compared to the untreated control, following fixing and staining the slides using methanol and Giemsa, respectively.

###  Cytotoxicity assay

 The present study used an MTT assay to investigate the cytotoxicity of prepared silver and dapsone niosomes and their combination in comparison with amphotericin B. For this purpose, the exposure of different concentrations (2.5–25 μg/mL) of each sample to the 5 × 10^5^ cells of murine macrophages was retained in 96- well microplates at 37 °C in 5 % CO_2_. Finally, after 72 hours treatment, the colorimetric MTT test was conducted using ELISA-reader as earlier defined in the anti-promastigote assay to determine the CC_50_. All the experiments were carried out in triplicate. Furthermore, the safety of prepared niosomes and their combination were compared to amphotericin B as positive control by measuring their selectivity index (SI) values based on the following equation:


(Eq. 2) $$SI\;\left(selectivity\;index\right)=CC50\;\left(macrophages\right)/IC50\;\left(amastigotes\right)$$

###  Flow cytometry assay

 In order to detect the apoptosis percentage of treated promastigotes, BD Pharningen^TM^ Apoptosis Detection kit of PI Annexin V was employed. For this purpose, after seeding 10^6^ promastigotes in a 2-mL microtube with RPMI-1640 medium enriched with 10 % v/v FBS, 100 µL of the different concentrations (5-100 μg/mL) of the prepared silver and dapsone niosomes combination and Amp B were added to the mixtures. Following the incubation of the samples at 25 °C, PBS was employed to wash the 72 hours treated promastigotes three times. Then, 5 µL of Annexin V and 7-AAD stain and 100 μL of binding buffer (1 × ) were added to each microtube, and they were stored in the dark at 25 °C for 20 minutes. Finally, a reading of the results and analysis of them were carried out using flow cytometry (Becton Dickinson) and Cell Quest software, respectively.

###  Quantitative real-time PCR 

 Quantitative real-time PCR was conducted to compare the expression levels of IFN-γ, IL-12p40, iNOS, TGF- β, and IL-10 gens in drug-exposed intra-macrophage amastigotes (Table 1). Initially, the Qiagen mini kit of RNeasy (CA, USA) was employed to extract total RNA based on its manufacturer’s instruction from intra-macrophage amastigotes. Consequently, they were exposed to different dilutions (10, 100, and 1000 μg/mL) of prepared niosomal formulation of silver (NA) and dapsone (ND), their combination (NA + ND), and Amp B as a drug of choice. Following the determination of the quality and concentration of RNA using NanoDrop spectrophotometer (ND-1000, Thermo Scientific, Wilmington, DE, USA), cDNA synthesis was performed by RT reagent kit (Prime Script^TM^, Takara, Japan). The process of reverse transcription was conducted for 20 minutes in a FlexCycler (Analytik Jena, Germany) at 37 °C using 0.5 μg of extracted RNA. In the next stage, Takara SYBR^®^ Premix Ex Taq^TM^ II (Japan) was used to obtain a total volume of 15 μL. Finally, the Qiagen Rotor-Gene Cycler system (USA) was employed to run the qPCR reaction in duplicate. Quantitative real-time PCR was carried out at 95 °C for 1 minute as a holding time and then, 40 three-step cycles of 95 °C for 15 seconds, 60 °C for 15 seconds, and 72 °C for 20 seconds, to conduct the denaturation, primer annealing, and extension, respectively, which was followed by 65 °C for 1 minute. 2 - ^ΔCt^ method was used to analysis qPCR data and obtain fold increase (FI) by comparing expression rates to the expression profile of Glyceraldehyde 3-phosphate dehydrogenase (GAPDH) as a reference gene.

**Table 1:** The sequences of using primers and reference gene (GAPDH)

**Gene**	**Forward sequence (5′–3′)**	**Reverse sequence (5′–3′)**	**Product size (bp)**
IL-12 P40	TGGTTTGCCATCGTTTTGCTG	ACAGGTGAGGTTCACTGTTTCT	171
IL-10	CTTACTGACTGGCATGAGGATCA	GCAGCTCTAGGAGCATGTGC	134
iNOS	ACATCGACCCGTCCACAGTAT	CAGAGGGGTAGGCTTGTCTC	89
TGF-β	CCACCTGCAAGACCATCGAC	CTGGCGAGCCTTAGTTTGGAC	112
IFN-γ	ACAGCAAGGCGAAAAAGGATG	TGGTGGACCACTCGGATGA	106
GAPDH	AGGTCGGTGTGAACGGATTTG	GGGGTCGTTGATGGCAACA	95

###  Antioxidant assay

 Evaluation of the antioxidant activity of dapsone and silver was carried out based on the 2, 2-diphenyl-1- picrylhydrazyl (DPPH) reduction as a free radical. The radical-scavenging activity of different concentrations (1-1000 μg/mL) of the samples was assessed in comparison with butylated hydroxyanisole (BHA) as positive control and DPPH solution without any treatment as untreated control.^30,31^ % DPPH radical scavenging was determined by reduction in drug-exposed DPPH solution absorbance using the following equation:


(Eq. 3) $$\%\;DPPH\;radical\;scavenging=\left(A_{C}–A_{S}/A_{C}\right)\times 100$$

Where A_C_ is the absorbance of untreated control and A_S_ is the absorbance of samples.

###  Molecular docking

####  iNOS protein practical residues investigation

 Utilizing the “Hotspot”^32^ and CASTp^33^ softwares we found the hotspots in the construction of iNOS regarding its function by bioinformatics databases.^34,35^

###  Pockets detection on the surface of iNOS protein

 It is extremely important to depict and specify external areas on3-dimensional (3-D) construction of proteins before docking. For this purpose, Molgro Virtual Docker (Molegro 2011) software is a practical tool, which is employed for a cavity search. Using this software pockets on the surface and cavities inside the structure of proteins are identifiable.

###  Protein-ligand docking

 Employing PubChem CID 2955^36^ and Protein Data Bank (PDB),^37^ we achieved the 3-D configuration of dapsone and iNOS. Molecular docking investigations were carried out in the restricted area around the potential binding sites using Molegro Virtual Docker software. Consequently, binding affinities were considered for choosing docking configurations and illustrating their graphs by Molegro Molecular Viewer 2.5.0 (Denmark).

###  Statistical analyses

 GraphPad Prism ver. 9.4 software (San Diego, USA) was employed to determine statistical differences between groups using one-way ANOVA and Tukey’s post hoc test. Measuring IC_50_ and CC_50_ values was conducted using a probit test in IBM SPSS Statistics 26 software (USA). Statistical values were considered significant at *P* < 0.05.

## Results

###  Characterization of niosomes

**Figure 1:**
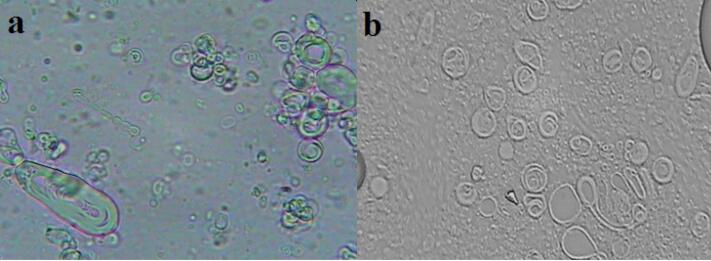
Light microscopic images (10 × 40 magnifications) of Span40/ Tween 40/ Cholesterol (35:35:30 m.r.) niosomes containing dapsone (a) and (1 % w/v) silver (b)

**Figure 2:**
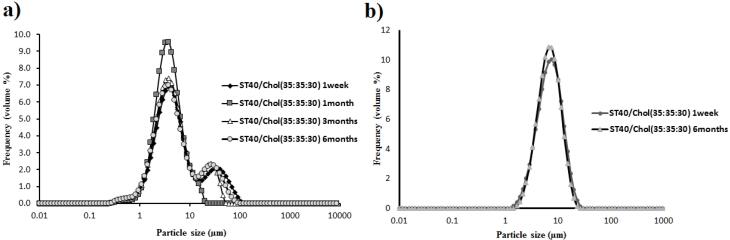
The particle size distribution graphs of Span40/Tween 40/Cholesterol (35:35:30 m.r.) formulations of dapsone (a) and silver (b) niosomes were measured at 1 week, 3, and 6 months after their preparation stored at 4–8 °C for up to 6 months

Figure 1 demonstrates the Span40/ Tween 40/ Cholesterol (35:35:30 m.r.) formulations of prepared niosomes. As shown in Figure 1, the polydispersity of vesicles is quite visible containing mostly multilamellar vesicles (MLVs) and large unilamellar vesicles (LUVs). Light microscopic images indicated a spherical shape and thin layer membrane of prepared nisosomes and the presence of no cholesterol crystal. The mean volume diameter of Span40/Tween 40/Cholesterol (35:35:30 m.r.) formulation of dapsone and silver niosomes was shown in Table 2 using DLS analysis. As can be seen from Figure 2, the prepared niosomes were in a narrow size range during a period of 6 months. The obtained results from DLS analysis revealed that no significant size alteration was observed, and vesicles remained in the nanometer range.

**Table 2:** evaluation of physical stability of prepared niosomes at defined time intervals (stored at 4-8 °C)

**Formulations**	**Vesicle size (µm), Mean±SD**				**Phase separation**	**Crystal formation**
	**1week**	**1 month**	**3 months**	**6 months**		
ST40/Chol (35:35:30) of dapsone niosomes	5.28 ± 0.30	4.16 ± 0.27	4.72 ± 0.23	5.00 ± 0.39	Not seen	Not seen
ST40/Chol (35:35:30) of silver niosomes	7.53 ± 0.06	7.59 ± 0.14	7.47 ± 0.03	7.34 ± 0.06	Not seen	A few

**Figure 3:**
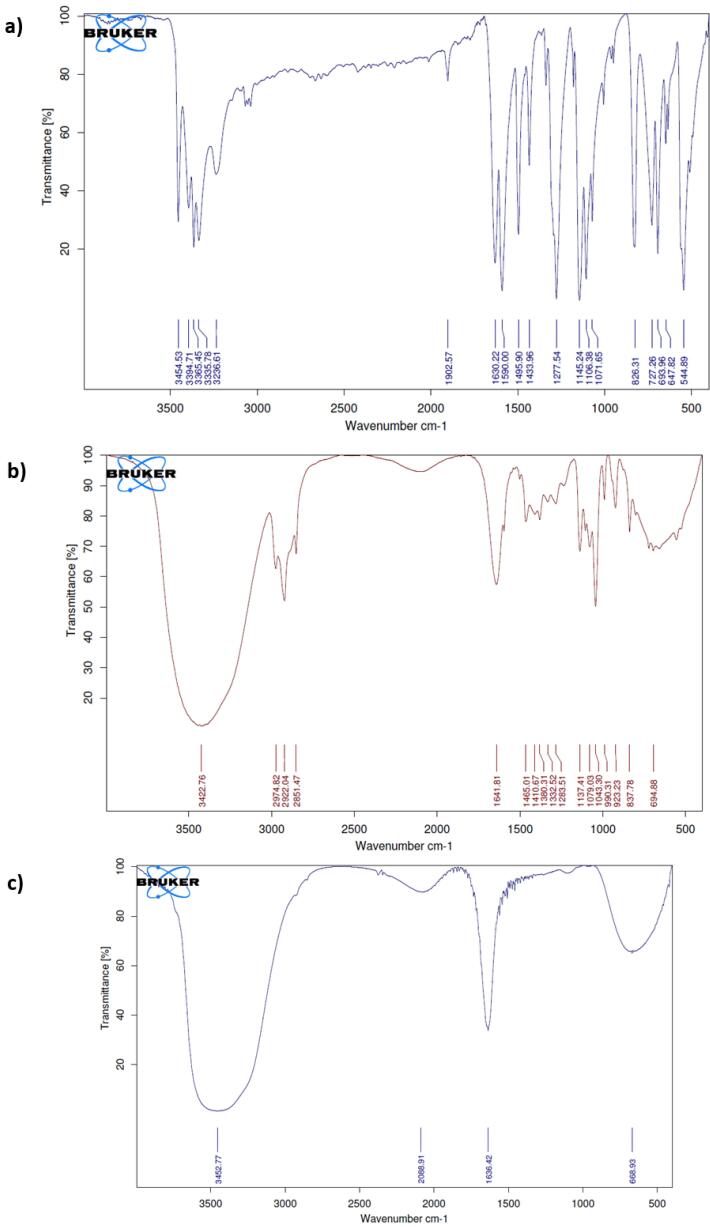
The FT-IR spectra of pure dapsone (a) and niosomal formulations containing dapsone (b) and silver (c)

Figure 3 (a, b, c) illustrates the FT-IR spectroscopy of pure dapsone and niosomal formulations of dapsone and silver, respectively. According to the FT-IR spectra of niosomal formulation containing dapsone, the strong absorption peak at 3422 cm^-1^ can be attributed to the O-H stretching vibration as the indicator of alcohol and phenol, and peaks in the region between 2800 to 3000 cm^-1^ are ascribed to the C-H stretching. It can be seen that a strong absorption peak at 1079 and 1043 cm^-1^ is corresponding to C-O bands and C-N (amines) stretch vibration, respectively. The FT-IR spectra of silver niosomes display OH bending vibration at 3452 cm^-1^. In addition, C = N and C = C stretching can be indicated due to the 1636 cm^-1^ band (Figure 3a, b, c).^38-41^

###  Encapsulation efficiency and in vitro drug release profile

**Figure 4:**
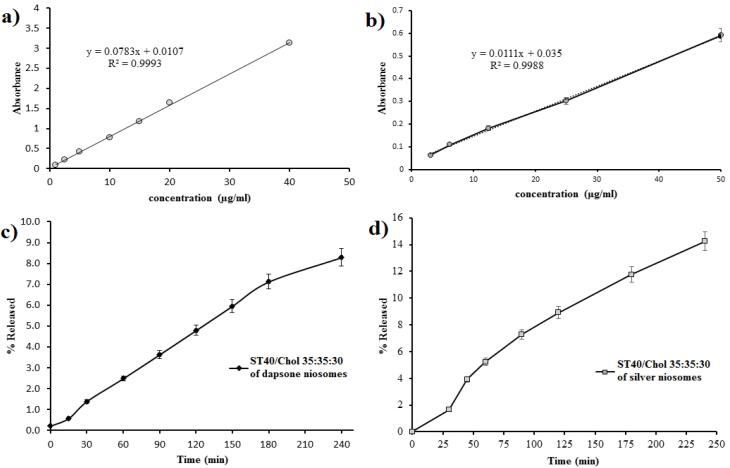
Standard calibration curve of dapsone (a), silver (b) and their *in vitro* release profiles of ST40/Chol 35:35:30 niosomal formulation (c, d)

 Encapsulation efficiency percentages (EE %) of active ingredients in Span40/Tween 40/ Cholesterol (35:35:30 m.r.) formulation during a period of 3 months are presented in Table 3. According to the results, dapsone encapsulation efficiencies were 96.77 ± 1.3 and 96.04 ± 0.87 on 1^st^ day and 3 months after preparation, respectively. Additionally, 68.5 ± 2.2 and 63.2 ± 1.7 of silver were incorporated into the vesicular formulation on 1^st^ day and 3 months after preparation, respectively. As can be seen from Figure 4, the obtained results from *in vitro* release study revealed that the release of both dapsone and silver from their niosomal formulations was controlled and prolonged. The *in vitro* release behavior of both active ingredients demonstrated that almost less than one percent of drugs were released from niosomes during the first 25 minutes. Furthermore, according to the diagrams, approximately 8 and 15 percent of dapsone and silver were released from their niosomal formulations over 240 minutes, respectively.

**Table 3:** Dapsone and silver encapsulation efficiency percent in their niosomal formulations on 1^st^ day and 3 months after preparation (mean ± SD, n = 3)

**Formulations**	**λ max (nm)**	**Mean EE %±SD 1st day**	**Mean EE %±SD 3 months**
ST40/Chol (35:35:30) of dapsone niosomes	260	96.77 ± 1.3	96.04 ± 0.87
ST40/Chol (35:35:30) of silver niosomes	424	68.5 ± 2.2	63.2 ± 1.7

###  Anti-intramacrophage amastigote effect

 As shown in Table 4, the mean number of amastigote form of *L. major *in 100 macrophages exposed to different concentrations of niosomal formulation of silver (NA) and dapsone (ND), their combination (NA + ND), and amphotericin B (Amp B) in comparison with the untreated control group. Results demonstrated a dose-dependent manner between increasing the concentration of samples and reduction in the number of amastigotes. There was a significant difference between Various concentrations of samples and the untreated control group (*P* < 0.001), and the least number of amastigotes (0.59 ± 0.62) was obtained for the highest concentration (100 μg/mL) of combination therapy (NA + ND). Furthermore, Table 5 displayed calculated IC_50_ values of tested compounds, and contrary to the anti-promastigote assay, results showed no significant difference between Amp B (3.30 ± 0.11 μg/mL) compared to ND (5.99 ± 0.48 μg/mL) and the combination of silver and dapsone niosomes (2.46 ± 0.34 μg/mL); however, an exception being for NA (8.52 ± 2.01 μg/mL) which was significantly less than that of Amp B (*P* < 0.01).

**Table 4:** Comparative assessment of the inhibitory effect of silver (NA) and dapsone (ND) niosomal formulation, their combination (NA + ND), and amphotericin B (Amp B) in comparison with untreated control against intramacrophage amastigotes of *L. major* after 72 h incubation.

**Concentrations** **(μg/mL)**	**NA**	**ND**
	**Mean±SD, ** ***P*** ** value **	**Mean±SD, ** ***P*** ** value**
0 (Untreated control)	41.12 ± 13.08, NR*	41.12 ± 13.08, NR
25	14.42 ± 3.35, *P* < 0.001	12.82 ± 2.38, *P* < 0.001
50	10.53 ± 2.83, *P* < 0.001	8.35 ± 2.29, *P* < 0.001
100	4.47 ± 1.23, *P* < 0.001	3.59 ± 1.18, *P* < 0.001
**Concentrations** **(μg/mL)**	**NA+ND**	**Amp B**
	**Mean±SD, ** ***P*** ** value **	**Mean±SD, ** ***P*** ** value**
0 (Untreated control)	41.12 ± 13.08, NR	41.12 ± 13.08, NR
25	10.65 ± 4.17, *P* < 0.001	10 ± 2.34, *P* < 0.001
50	4.29 ± 3.23, *P* < 0.001	6.12 ± 1.45, *P* < 0.001
100	0.59 ± 0.62, *P* < 0.001	2.29 ± 1.31, *P* < 0.001

* Not related

**Table 5:** Comparative assessment of the mean IC_50_ values of niosomal formulation of silver (NA) and dapsone (ND), their combination (NA + ND), and amphotericin B (Amp B) as positive control against promastigote and amastigote forms of *L. major* and CC_50_ values of them on J774-A1 cell line using selectivity index (SI)

**Drugs**	**Amastigote**	**Promastigote**	**Macrophage**	**SI**
	**IC**_50_ ** (µg /mL), ** ***P*** ** value**	**IC**_50_ ** (µg /mL), ** ***P*** ** value**	**CC**_50_ ** (µg /mL), ** ***P*** ** value**	**CC**_50_ **/IC**_50 _
Amp B	3.30 ± 0.11, NR*	3.99 ± 0.64, NR	24.53 ± 1.18, NR	7.43
NA	8.52 ± 2.01, P < 0.01	12.84 ± 0.63, P < 0.001	46.80 ± 3.66, *P* < 0.001	5.49
ND	5.99 ± 0.48, ns	9.87 ± 0.74, P < 0.001	40.61 ± 2.95, *P* < 0.01	6.78
NA + ND	2.46 ± 0.34, ns	2.35 ± 0.37, P < 0.05	43.32 ± 4.53, *P* < 0.001	17.61

* Not related.

###  Anti-promastigote effect

**Figure 5:**
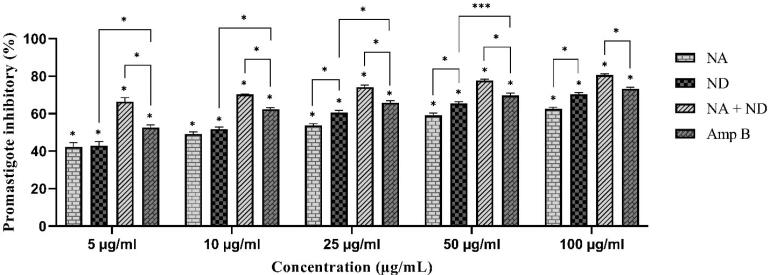
The total mean inhibitory values of *L. major* promastigotes exposed to different concentrations of niosomal formulation of silver (NA) and dapsone (ND), and their combination (NA + ND) compared to amphotericin B (Amp B) as a positive control group after 72 h incubation. Bars display the mean ± SD of inhibitory values of triplicate experiments (*P < 0.001, **P < 0.01, ***P < 0.05).

Figure 5 presents the mean inhibitory percentage of promastigote forms of *L. major* parasites exposed to different dilutions of niosomal formulation of silver (NA) and dapsone (ND), and their combination (NA + ND) compared to amphotericin B (Amp B) as a positive control group. The obtained results demonstrated a dose-dependent response for all the samples. *L. major *promastigote proliferation rate was inhibited significantly (*P < 0.001) in each concentration of samples compared to the negative control group. Although the inhibitory percentage of the parasites exposed to Amp B was significantly higher than NA (*P < 0.001) and ND alone (at all the concentrations, except 100 µg/mL) (*P < 0.001, ***P < 0.05), the superior inhibitory effect was obtained for the combination of silver and dapsone niosomes in comparison with Amp B (*P < 0.001). Additionally, ND samples displayed significantly higher antiproliferation effects against *L. major *promastigotes compared to NA at higher concentrations (25, 50, and 100 µg /mL) (*P < 0.001). As shown in Table 5, there is a significant difference between calculated IC_50_ values of Amp B (3.99 ± 0.64 µg /mL) and NA (*P < 0.001), ND (*P < 0.001), and their combination (***P < 0.05) samples against promastigotes of *L. major*, which were 12.84 ± 0.63, 9.87 ± 0.74, and 2.35 ± 0.37, respectively.

###  Isobolographic analysis

**Figure 6:**
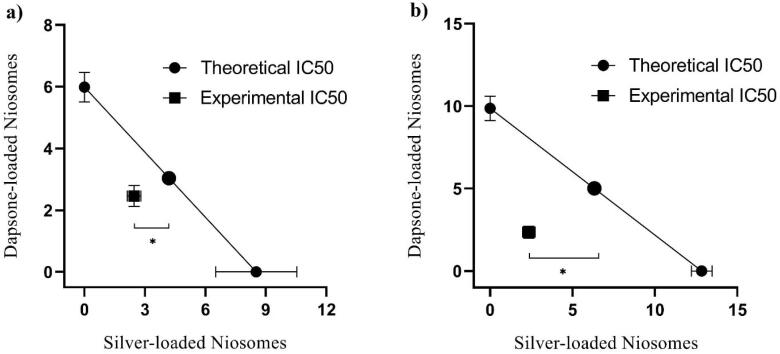
The isobologram analysis of leishmanicidal activities of silver-loaded and dapsone-loaded niosomes against intramacrophage amastigotes (a) and promastigotes (b) of *L. major.* The oblique line in both figures is the theoretic additive effect line, and the focus under the line is the experimental IC_50_ of the niosomal combination co-administration. Bars display the mean ± SD of triplicate experiments (*P < 0.001)

 The isobolographic analysis was employed to assess the silver-dapsone interaction by comparing the theoretical and experimental IC_50_ of their niosomal formulations co-administration (Figure 6). After calculating the theoretical IC_50_ of niosomes combinations using the following equation (Eq. 4), a one-sample *t*-test was used to determine the statistical difference between theoretical and experimental IC_50_, which was significant (*P < 0.001).^42^


(Eq. 4) $$Theoretical\;IC_{50}=\left(IC_{50\;Silver}/2\right)+\left(IC_{50\;Dapsone}/2\right)$$

 Moreover, the combination index (CI) the other most popular method for investigating drug interaction in combination chemotherapy was measured by the following equation (Eq. 5), in which the CI value quantitatively defines synergistic (CI < 1), additive (CI = 1) and antagonist (CI > 1) interaction^43^:


(Eq. 5) $$CI=\left(IC_{50\;Mix}\right)/\left(IC_{50\;Silver}\right)+\left(IC_{50\;Mix}\right)/\left(IC_{50\;Dapsone}\right)$$

The results of both isobologram and CI (0.7 for amastigote assay and 0.42 for promastigote assay) analyses revealed a synergistic effect between silver-loaded and dapsone-loaded niosomes upon induction of leishmanicidal effect against intramacrophage amastigote and promastigote forms of *L. major*.

###  Cytotoxicity effect

**Figure 7:**
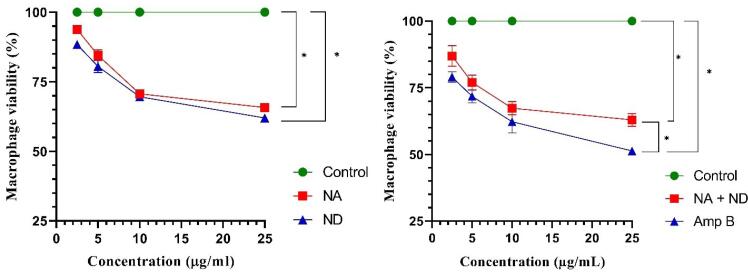
viability rate of murine macrophage cells (J774-A1) treated with different concentrations of niosomal formulation of silver (NA) and dapsone (ND), their combination (NA + ND), and amphotericin B (Amp B) in comparison with an untreated control group using MTT assay. Bars display the mean ± SD of inhibitory values of triplicate experiments (*P < 0.001)

Figure 7 indicates the obtained results of the viability rate of murine macrophage cells (J774-A1) treated with different concentrations of niosomal formulation of silver (NA) and dapsone (ND), their combination (NA + ND), and amphotericin B (Amp B) in comparison with an untreated control group using MTT assay. Significant cytotoxicity (*P* < 0.001) in macrophage cell line was observed for all the concentrations of samples except 2.5 μg/mL concentration of NA. Results displayed no significant difference in the cytotoxicity effect of J774 A1 cells between NA and ND. Whereas, the viability rates of macrophages exposed to 2.5 and 25 μg/mL samples of NA and ND combination were significantly higher than Amp B (*P* < 0.01, *P* < 0.001). Comparing calculated CC_50_ values of using drugs revealed that there was a significant difference between prepared niosomes and their combination compared to Amp B as a positive control (*P* < 0.001, *P* < 0.01) (Table 5). According to this, NA, ND, and their combination exhibited a lower cytotoxicity effect against the murine macrophages in comparison with Amp B.

 As can be seen in Table 5, the selectivity index (SI) values for niosomal formulation of silver (NA) and dapsone (ND), their combination (NA + ND), and amphotericin B (Amp B) were measured based on the previous equation (Eq. 2), which were 5.49, 6.78, 17.61, and 7.43, respectively.

###  Antioxidant activity

**Figure 8:**
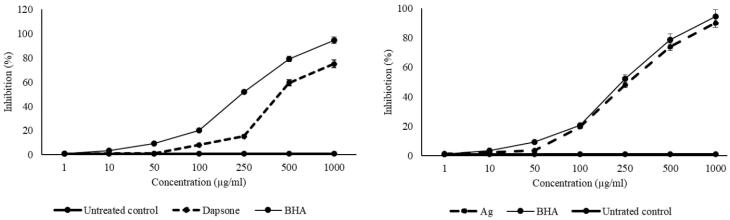
DPPH free radical scavenging activity of niosomal formulation of silver and dapsone in comparison to the butylated hydroxyanisole (BHA) as a positive control. Bars display the mean ± SD of triplicate experiments

 Evaluating the hydrogen donations from niosomal formulation of dapsone and silver determined the radical-scavenging activity of these compounds against DPPH in comparison with an untreated control group and BHA as a positive control (Figure 8a, b). Results displayed a dose-response effect for all the components. Based on the overall IC_50_ values, the scavenging effects of dapsone-loaded (648.6 µg/mL) and silver-loaded niosomes (249.8 µg/mL) against DPPH were significantly lower than BHA (206.15 ± 0.78 µg/mL) (*P* < 0.001) but within an acceptable range.

###  Apoptotic analysis

**Figure 9:**
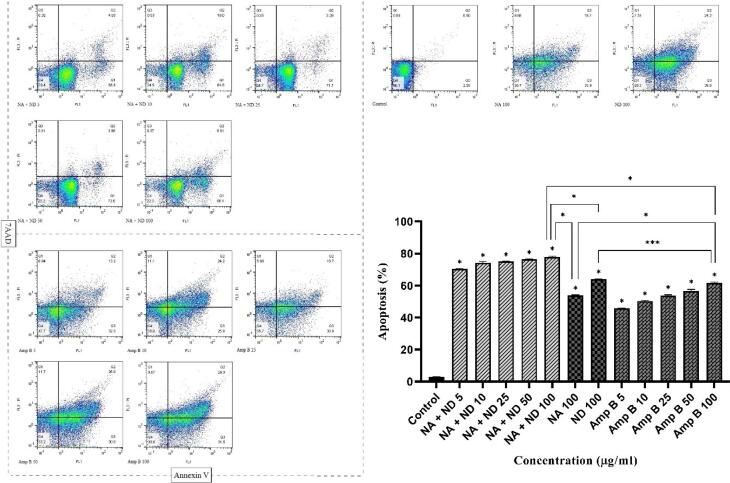
Early and late apoptotic values of *L. major *promastigotes exposed to various concentrations of silver and dapsone niosomal combination in comparison with untreated control and amphotericin B as the positive control group. Bars display the mean ± SD of triplicate experiments (*P < 0.001, **P < 0.01, ***P < 0.05)

 Employing a flow cytometry assay after staining samples with annexin V/propidium iodide (PI) revealed that apoptosis can be considered as one of the mechanisms of cell death triggered by niosomal formulations of silver and dapsone. Based on our findings, all concentrations of silver and dapsone niosomes, their combination, and amphotericin B induced the death of *L. major* promastigotes significantly by programmed cell death in a dose-dependent manner (*P < 0.001). According to Figure 9, the amount of both early (stained only with Annexin V (Annexin V + /PI−), bottom right) and late apoptotic cells (stained with both Annexin V and PI (Annexin V + /P + ), top right) was considered as the proportion of apoptosis.^44,45^ Based on the measured apoptotic values, there was a significant difference between a similar amount of niosomal combination and Amp B (*P < 0.001). Besides, the maximum rate of apoptosis (77.31%) was observed when *L. major *promastigotes were exposed to 100 µg /mL of niosomal combination, which was higher than apoptotic values of each niosomes alone at this concentration (*P < 0.001).

###  Gene expression

**Figure 10:**
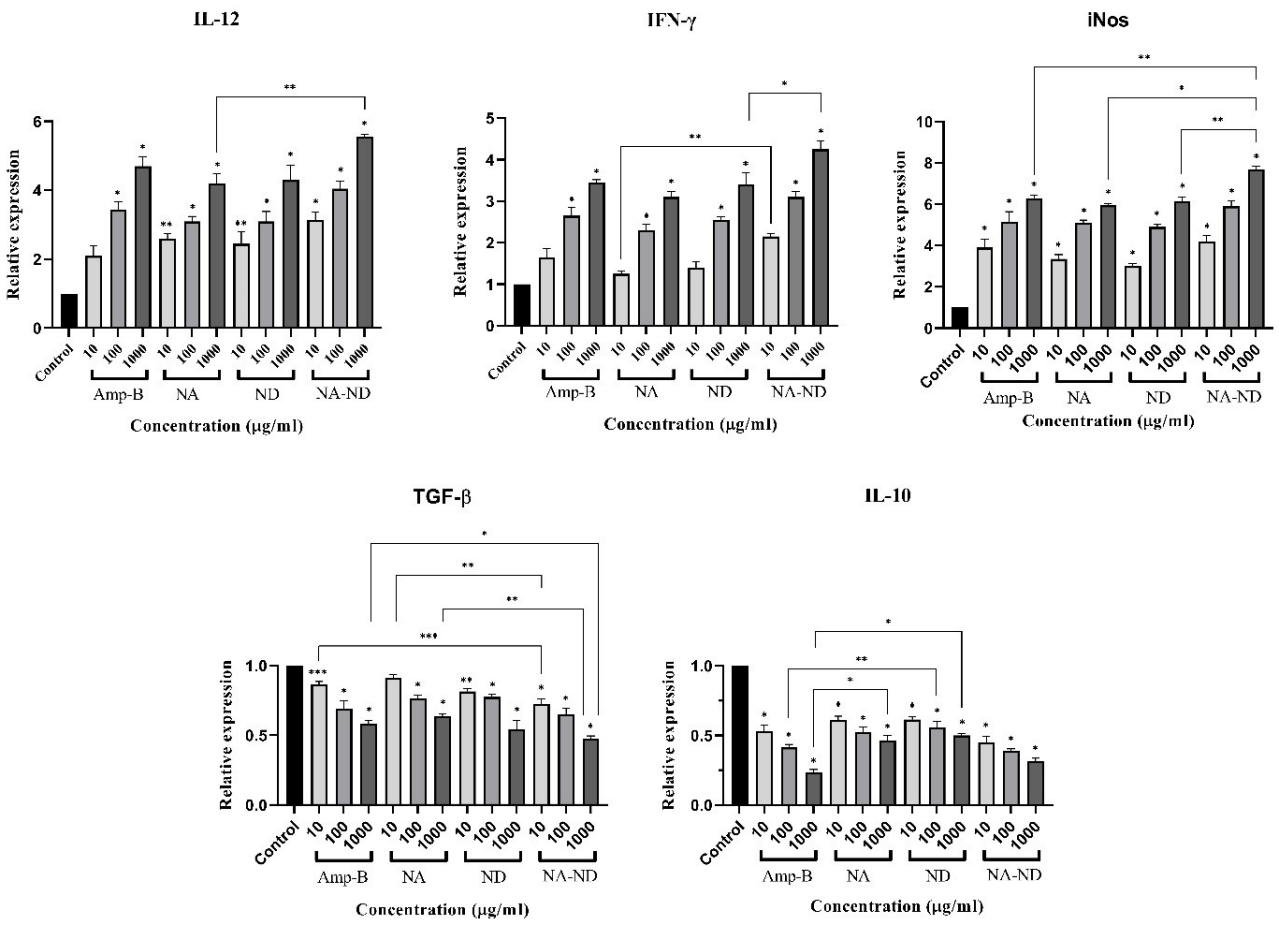
The gene expression levels of IFN-γ, IL-12p40, and iNOS (Th1 cell-related parameters) and IL-10 and TGF-β (Th2 cell-related parameters) of macrophages exposed to various concentrations of amphotericin B, silver-loaded and dapsone-loaded niosomes alone, and their niosomal combination compared to the untreated group. Bars display the mean ± SD of duplicate experiments (*P < 0.001, **P < 0.01, ***P < 0.05)

 The analysis of T cell-mediated immune responses was conducted by evaluating the related gene expression levels. For this purpose, expression profiles of IFN-γ, IL-12p40, and iNOS (Th1 cell-related parameters) and IL-10 and TGF-β genes (Th2 cell-related parameters) of treated macrophages with amphotericin B, silver-loaded and dapsone-loaded niosomes alone, and their niosomal combination were compared to the reference gene (Figure 10). The expression levels of Th1-related cytokines revealed a significant increase in high concentrations of samples compared to the untreated group, whilst down-regulated expression of Th2-related transcription factors was observed from 10 to 1000 µg /mL (*P < 0.001, **P < 0.01, ***P < 0.05). Regarding Th1 cell-related parameters, there was no significant difference between niosomal combination and amphotericin B at the same concentrations except for 1000 µg /mL samples in iNos expression (**P < 0.01). As can be seen in Th2 cell-related expression profiles, significant levels were obtained in TGF-β expression between niosomal combination and amphotericin B at the concentration of 10 and 1000 µg /mL (*P < 0.001, ***P < 0.05). In contrast, the rates of IL-10 expression in the combination group were similar to the positive control.

###  Molecular docking

**Figure 11:**
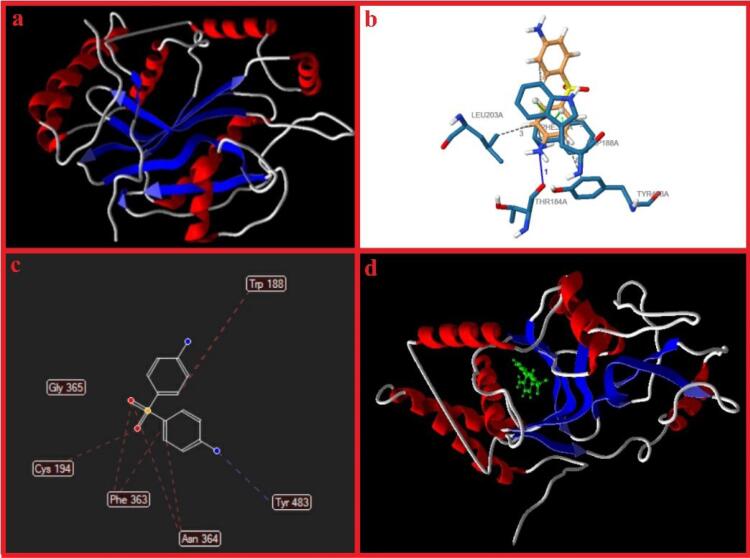
iNOS protein surface configuration (a), anticipated amino acids in pocket arrangement using PLIP online server, and results of molecular docking obtained by Molegro Virtual Docker software (c, d)

 The structural and functional activity of the iNOS protein surface is displayed in Figure 11a. Ligand Interaction Profiler (PLIP) was employed on an online server to identify the amino acids included within the interplay between iNOS and dapsone. Following that, the 2-D interaction graphs demonstrated profoundly hydrophobic cavities comprising several ligand-cavity hydrogens and hydrophobic interactions as the most contributing powers in the ligand-protein binding energy. Based on steric interactions, Figure 11b showed the interactions between dapsone and amino acids of iNOS protein.

 Results of molecular docking revealed that dapsone binds to iNOS (Figure 11c, d) with the active site residues, which are reported in Table 6. Moreover, the MolDock score was 109.575 kcal/mol (Table 7).

**Table 6:** Contribution of the iNOS residues

**Hydrophobic Interaction**								
**Index**		**Residue**		**AA**	**Distance**	**Ligand Atom**	**Protein Atom**	
1		188A		TRP	2.96	2944	735	
2		188A		TRP	3.71	2947	728	
3		203A		LEU	3.47	2945	898	
4		363A		PHE	3.70	2945	2317	
5		363A		PHE	3.35	2943	2321	
6		483A		TYR	3.96	2947	2762	
**Hydrogen Bonds**								
**Index**	**Residue**		**AA**	**Distance H-A**	**Distance D-A**	**Donor Angle**	**Donor Atom**	**Acceptor Atom**
1	184A		THR	2.93	3.73	136.98	2937 [Npl]	690 [O2]
**π-Stacking**								
**Index**	**Residue**	**AA**	**Distance**	**Angle**	**Offset**	**Stacking Type**	**Ligand Atoms**	
1	188A	TRP	3.79	21.46	0.12	P	2939, 2941, 2943, 2945, 2947, 2949	
2	188A	TRP	4.32	20.82	0.82	P	2939, 2941, 2943, 2945, 2947, 2949	

**Table 7:** Molecular docking score

**Type**	**Heavy atoms**	**total**	**ELntra**	**Epair**
All atoms	17	-109.575	-11.1642	-120.739

## Discussion

 Genus *Leishmania*, as an intracellular parasite, causes a vector-borne neglected tropical infection named leishmaniasis.^46^ Currently, there is a concern in over 90 countries worldwide regarding increasing resistance among *Leishmania* species.^47^ Therefore, researchers and global health agencies are forced to develop new drugs and seek new strategies to overcome this zoonotic disease. Combination or multi-drug administration and nano-based drug delivery systems can be considered the main strategies for the treatment of leishmaniasis as alternative therapies. This study aims to explore combination therapy and nanostructured lipid carriers simultaneously to obtain the efficient delivery and combat side effects of conventional antileishmanial regimens.^48,49^ Niosomes as the circular bilayer nanometric size vesicles, contain non-ionic surfactants, which reduce the systemic non-selective toxicity and reticular-endothelium system elimination of their entrapped drugs.^50^

 Several studies have reported the anti-inflammatory, antimicrobial, and anti-leishmaniasis effects of dapsone, however, serious side effects limit its use.^51^ The findings of recent clinical trial demonstrated higher anti-leishmaniasis efficacy of dapsone niosomal gel and intralesional meglumine antimoniate combination in comparison with standard treatment, which was cryotherapy combined with intralesional meglumine antimoniate.^52^ The same research team later suggested that niosomal DAP gels can be considered an alternative therapy to cryotherapy in the treatment of cutaneous leishmaniasis.^53^ On the other hand, previous studies have confirmed the leishmanicidal activities of Ag-NPs as promising antileishmanial agents, which cause several immune responses such as inducing programmed cell death and generation of reactive oxygen species.^54-56^ Therefore, the film hydration method was employed to prepare different unique niosomal formulations of dapsone and silver to study the anti-parasitic effect of their combination compared to silver-loaded and dapsone-loaded niosomes alone against *L. major*. For this purpose, after conducting physicochemical studies, Span 40 and Tween 40/cholesterol with a 7/3 molar ratio was selected as the optimal composition niosomes. The results of morphological observations and laser diffraction particle size assays displayed low particle size and narrower size distribution of prepared niosomes. Besides, the formation of final niosomal formulations and the characterization of their functional group was studied using FT-IR spectroscopy, which confirmed a strong linkage between niosomal structures and their incorporated active agents. Our findings indicated high stability, controlled release pattern, and acceptable values of entrapment efficacy for silver and dapsone niosomal formulations.

 To the best of our knowledge, there is no reported study surveying the combined potential of dapsone and silver-loaded niosomes against protozoan parasites of *L. major*. The observed antiproliferative effects showed lower antileishmanial efficiency of silver-loaded and dapsone-loaded niosomes alone in comparison with reference drug amphotericin B, however, their combination was more potent against both forms of intracellular amastigotes and promastigotes. Besides, the results of isobologram and CI analyses confirmed the synergic potential of this niosomal combination. Similarly, other studies displayed further improvement in combination therapy’s leishmanicidal activity.^10,57^ The present study showed that the combination of multidrug therapy and nano-based drug delivery systems could be considered a promising strategy to enhance treatment efficacy and decrease the development of resistance.

 Despite studying the antiparasitic activity of prepared niosomal formulations, this study aims to obtain a deeper understanding of their possible mechanisms of action to discover new therapeutic targets. Establishing a flow cytometry assay to identify the apoptotic characteristics of prepared niosomes revealed the synergistic effect of niosomal combination therapy to induce the apoptotic pathways as the main mechanism of parasite cell death. Flow cytometry findings indicated the induction of early and late apoptosis in *L. major* promastigotes for silver and dapsone niosomal combination and amphotericin B in a dose-dependent manner.

 The findings of antioxidant assay indicated the radical scavenging properties of silver- and dapsone-loaded niosomes as one of the potential mechanisms of action against *Leishmania* parasites.^58^ The mechanism of action is based on the increased generation of reactive oxygen species, which plays an essential role against parasitic infections like leishmaniasis.^59,60^

 Evaluating the gene expression levels of Th1 and Th2 cells were designed to clarify the action mode of the prepared niosomes. Th1 response is essential to anti-parasite immune responses in potentiating macrophage activation and parasite death.^61^ iNOS is activated by Th1/IFN-γ-induced signaling in infected macrophages resulting in NO generation.^62,63^ Furthermore, NK cells could be stimulated in reply to Th1 immune responses such as IL-12p40 signaling.^64^ As mentioned before, these cytokines directly or indirectly are related to the removal of the intramacrophage amastigote stage.^65^ These immunoregulatory functions were substantially increased when silver-loaded niosomes were combined with dapsone-loaded niosomes. Conversely, these Th1 responses could be inhibited by TGF-β and IL-10 cytokines, leading to macrophage inactivation.^66^ Suppression of innate and adaptive cell responses could also be another immunomodulatory potential of these cytokines, inhibiting inflammatory cell function via the downregulation of Th1 cell-related genes.^67^ The progression of these Th2 cytokines is involved with parasites’ persistence and replication, resulting in susceptibility to *L. major* infection by inhibiting the stimulation and maturation process of macrophages.^68^ Monitoring the cytokines associated with the Th2 phenotype showed a significant decrease after treating the infected macrophages with prepared niosomes and their combination.

 Determining known bioactive compounds’ interplays with their potential biomolecular targets is vital for advancing new therapies. Therefore, the field of *in silico* prediction is productive in the treatment development process.^69^ So the molecular docking simulating and calculating conducted for selected active gene iNOS revealed that dapsone could occupy the active sites via various chemical bond interactions with high binding energy.

## Conclusion

 Our overall results strongly suggest that the co-administration of dapsone niosomes and silver niosomes has a synergistic effect against *L. major* parasites *in vitro*. Moreover, these prepared niosomes demonstrated more destructive action when combined by inducing apoptosis in the parasite and triggering antileishmanial pathways of macrophages. Our findings confirmed one of the objectives of an ideal drug combination, attenuating drug toxicity to the host cells. Comparing calculated selectivity index of prepared niosomes and their combination displayed that this combination has a lower cytotoxic effect on mammalian macrophages compared to their niosomes alone. Based on the above results, the multifunctional leishmanicidal activity of dapsone- and silver-niosomes co-administration advocates this combination as a promising alternative therapy for the treatment of cutaneous leishmaniasis. However, further *in vivo* and clinical studies are needed to determine the toxicity parameters of these compounds in various populations of volunteer patients with cutaneous leishmaniasis.

## Competing Interests

 There are no conflicts to declare

## Ethical Approval

 Not applicable.
